# Metagenomics of pasteurized and unpasteurized gouda cheese using targeted 16S rDNA sequencing

**DOI:** 10.1186/s12866-018-1323-4

**Published:** 2018-11-19

**Authors:** Joelle K. Salazar, Christina K. Carstens, Padmini Ramachandran, Arlette G. Shazer, Sartaj S. Narula, Elizabeth Reed, Andrea Ottesen, Kristin M. Schill

**Affiliations:** 1 Division of Food Processing Science and Technology, Office of Food Safety, U. S. Food and Drug Administration, Bedford Park, IL USA; 2 Division of Microbiology, Office of Regulatory Science, U. S. Food and Drug Administration, College Park, MD USA; 3 Illinois Institute of Technology, Institute for Food Safety and Health, Bedford Park, IL USA

**Keywords:** Metagenome, 16 s rDNA, Cheese, Dairy, Gouda, Unpasteurized milk

## Abstract

**Background:**

The microbiome of cheese is diverse, even within a variety. The metagenomics of cheese is dependent on a vast array of biotic and abiotic factors. Biotic factors include the population of microbiota and their resulting cellular metabolism. Abiotic factors, including the pH, water activity, fat, salt, and moisture content of the cheese matrix, as well as environmental conditions (temperature, humidity, and location of aging), influence the biotic factors. This study assessed the metagenomics of commercial Gouda cheese prepared using pasteurized or unpasteurized cow milk or pasteurized goat milk via 16S rDNA sequencing.

**Results:**

Results were analyzed and compared based on milk pasteurization and source, spatial variability (core, outer, and under the rind), and length of aging (2–4 up to 12–18 months). The dominant organisms in the Gouda cheeses, based on percentage of sequence reads identified at the family or genus levels, were *Bacillaceae*, *Lactococcus*, *Lactobacillus*, *Streptococcus*, and *Staphylococcus*. More genus- or family-level (e.g. *Bacillaceae*) identifications were observed in the Gouda cheeses prepared with unpasteurized cow milk (120) compared with those prepared with pasteurized cow milk (92). When assessing influence of spatial variability on the metagenomics of the cheese, more pronounced differences in bacterial genera were observed in the samples taken under the rind; *Brachybacterium*, *Pseudoalteromonas*, *Yersinia*, *Klebsiella*, and *Weissella* were only detected in these samples. Lastly, the aging length of the cheese greatly influenced the number of organisms observed. Twenty-seven additional genus-level identifications were observed in Gouda cheese aged for 12–18 months compared with cheese only aged 2–4 months.

**Conclusions:**

Collectively, the results of this study are important in determining the typical microbiota associated with Gouda cheese and how the microbiome plays a role in safety and quality.

## Background

High-throughput metagenomic sequencing technology has transformed the ecological study of food products. Targeted metagenomics utilizes gene fragment DNA sequencing to determine identities of microbiota such as bacteria, yeast and mold. In most cases, a conserved segment of a hypervariable region of the 16S rDNA gene is used. In recent years, the field of metagenomics has flourished and published studies now include insights into the microbiomes of cilantro [1], spinach [2], bean sprouts [3], kimchi [4], kefir [5], meat [6], wine [7], and cheese [8–13]. The composition of the native microbiota in these food products may help determine property characteristics contributed to by microorganisms such as flavor, texture, color, aroma, shelf-life, and spoilage.

Cheese is composed of microorganisms, originating from the raw ingredients used, the environment, and added starter cultures as well as adjunct cultures. These many sources of microbes cause considerable variability in the microbiome across cheese varieties. Starter cultures, generally comprised of lactic acid bacteria, aid in acidification and fermentation during cheese production and, in some instances, are replaced by more competitive organisms during brining, rind development, and aging [14]. Other microorganisms, such as fungi, are integral to the manufacture of smear-ripened cheeses such as Reblochon and Taleggio. Metagenomics can aid in understanding the microbiota of cheese, how these organisms interact, and how the presence of certain organisms aid or hinder aspects of food quality and safety. For example, it is known that certain microbial consortia in cheese contain antilisterial properties [15–17]. Studies have assessed the microbiomes of various cheeses inoculated with *Listeria monocytogenes* and determined that the pathogen was inhibited by a combination of lactic acid bacteria and Gram-positive, catalase-positive bacteria [17]. Ultimately, the identification of the microbial communities in cheese is important and must include how factors such as raw materials, aging, storage conditions, and specific product characteristics impact diversity.

Manufacturing of cheeses is divided into several steps. The first step is the addition of starter cultures which acidify the milk during the ripening process. The milk and starter culture mixture is subsequently warmed prior to the addition of rennet, leading to curd formation and whey separation. The resulting curd is cut, cooked, and placed in molds that are pressed into the desired shape. The finished cheese wheels are brined and aged depending on the cheese type being processed. Cheeses can be manufactured using either pasteurized or unpasteurized milk. The pasteurization of milk involves heat treatment to eliminate harmful pathogens and lower the overall microbial burden. Therefore, cheeses prepared using unpasteurized milk are generally comprised of more diverse and heterogeneous microorganisms. Previous research has determined that the native microbiota in unpasteurized milk contributes to the sensory properties of the resulting cheese, and research suggests that these organisms aid in producing a more robust flavor [18, 19]. The microbiota in unpasteurized milk is influenced by the animal’s teat canal and surrounding skin, the environmental conditions, seasonality, pasture and grazing changes, personnel hygiene, starter culture selection, process and post-process contamination [14, 20–22]. For pasteurized milk, the microbial community is influenced by the thermoduric bacteria that survive pasteurization, and post-process contamination.

The composition and distribution of microbiota in cheeses differ not only by milk type and environmental factors, but also by sampling location (i.e., core, rind) [8, 16, 21, 23]. The rind of the cheese is a more open ecosystem exposed to the environment and abiotic conditions and is comprised of a high diversity of organisms [21]. Aerobic bacteria, as well as yeasts and molds, dominate the rind of cheese. Halophiles are also present due to their ability to survive high salt concentrations that would be encountered during brining. Hygienic conditions during aging also play a role in shaping the microbiota of the rind. Conversely, the core of the cheese is more anaerobic and generally exhibits a lower pH, leading to less biodiversity [21]. For this reason, lactic acid bacteria, including those in the added starter cultures, are predominant in the core, often reaching population levels near 9 log CFU/g shortly after cheese manufacture [21]. These bacteria acidify the milk and hinder the growth of other less-competitive species and some spoilage bacteria. Less dominant organisms in the core include yeasts, Gram-positive catalase-positive bacteria, and enterococci. These different microenvironments encountered throughout cheese affect the types of microbiota present and how these organisms interact.

Cheeses can be categorized on their degree of firmness of texture and are grouped as hard, semi-hard and soft. Hard and semi-hard cheeses are often aged. For aged cheeses, such as Gouda, Cheddar, and Parmesan, the length of the aging process plays a significant role in the composition and diversity of microbiota. When cheeses are aged, moisture content and water activity decrease. Due to increasingly limited nutrients during aging, some bacteria undergo autolysis, contributing cellular components, including enzymes and sugars, to the overall characteristics of the cheese [24]. It is known that populations of starter lactic acid bacteria are reduced but survive during aging. In the U. S., cheeses crafted using unpasteurized milk must be aged for at least 60 days at a minimum temperature of 35 °F (1.67 °C) prior to introduction into interstate commerce [25]. This aging period is intended to eliminate pathogens that may have been present in the unpasteurized milk. However, it has been determined that pathogens can survive past the 60 day aging process [26–28]. Understanding the microbiome of Gouda, an aged cheese, will aid in designing studies to reduce the risk of illness due to pathogens from consumption of this type of cheese made using unpasteurized milk.

During 1998–2015 a total of 113 outbreaks associated with cheese consumption were reported to the CDC, resulting in 2418 illnesses, 291 hospitalizations, and 18 deaths [CDC FOOD Tool, wwwn.cdc.gov/foodborneoutbreaks]. Of the total number of outbreaks, 20% (*n* = 23) were specifically stated to be associated with cheeses made using unpasteurized milk. Gouda cheese has frequently been implicated in product recalls and outbreaks [29–31] attributed to various foodborne pathogens including *Listeria monocytogenes* and *E. coli* O157:H7. The goal of this study was to determine the baseline microbiota associated with Gouda cheese via 16S rDNA metagenomic sequencing. Gouda cheese in particular was selected as the model product because it is an aged cheese that is required to be held at ≥35 °F for at least 60 days if manufactured from unpasteurized milk in order to ensure product safety. Variables examined in this study included milk type (i.e. unpasteurized, pasteurized), milk origin (i.e. bovine, caprine), aging duration (from 2 to 4 to 12–18 months), and sampling location (i.e. inner or outer cheese). Elucidation of the native microbiota of Gouda cheese will allow estimation of product quality potential and overall safety.

## Results

### Composition analysis of commercial gouda cheese

In this study, Gouda cheese samples were analyzed for moisture, salt, fat, pH, and a_w_ to assess variations in these physical property characteristics (see Tables 1 and 2). All cheese samples met the CFR requirement for moisture content (maximum of 45%) [25], however a wide range of values were determined: 18.06 (brand C, under the rind) to 42.41% (brand A, under the rind). The Gouda cheeses made with goat milk (F-H) had the highest fat in solid content: 51.62–55.91%. Fat content ranged from 43.09 (brand D) to 55.91% (brand G). Brands D, I, K, and N had slightly lower fat in solid content than the 45% minimum specified in the CFR, ranging from 43.09–44.25%.

**Table 1:** Composition of the pasteurized Gouda cheese samples

Brand and location	pH	a_w_	Moisture (%)	Salt (%)	Fat in solids (%)
A					
Under the rind	6.37	0.954	42.41	2.31	ND
Core	5.26	0.957	41.23	2.39	ND
Inside	5.41	0.957	41.79	2.39	47.24
B					
Under the rind	5.76	0.950	31.24	2.04	ND
Core	5.43	0.948	33.16	2.15	ND
Inside	5.44	0.951	37.44	2.26	47.15
C					
Under the rind	5.75	0.877	18.06	1.49	ND
Core	5.64	0.895	20.63	1.69	ND
Inside	5.71	0.902	25.92	2.00	49.95
D					
Under the rind	6.02	0.931	25.98	1.21	ND
Core	5.86	0.936	31.56	1.44	ND
Inside	5.85	0.931	26.89	1.34	43.09
E					
Under the rind	5.97	0.938	30.36	2.08	ND
Core	5.59	0.937	32.69	2.10	ND
Inside	5.44	0.937	33.82	2.29	49.86
F					
Under the rind	5.53	0.908	21.38	1.65	ND
Core	5.39	0.913	24.50	1.70	ND
Inside	5.40	0.920	26.80	2.00	53.96
G					
Under the rind	5.83	0.923	27.52	1.77	ND
Core	5.40	0.939	32.53	1.98	ND
Inside	5.34	0.940	35.61	2.21	55.91
H					
Under the rind	5.84	0.943	29.38	1.71	ND
Core	5.50	0.943	32.24	1.79	ND
Inside	5.64	0.944	33.16	1.87	51.62

Brands A through E were prepared using cow milk and brands F through H were prepared using goat milk

**Table 2:** Composition of the unpasteurized Gouda cheese samples

Brand and location	pH	a_w_	Moisture (%)	Salt (%)	Fat in solids (%)
I					
Under the rind	6.29	0.936	23.10	1.43	ND
Core	5.34	0.927	26.35	1.69	ND
Inside	5.27	0.940	31.08	1.81	44.25
J					
Under the rind	5.79	0.902	19.69	1.71	ND
Core	5.50	0.902	21.64	2.00	ND
Inside	5.57	0.903	24.29	2.25	50.85
K					
Under the rind	5.75	0.941	26.93	1.42	ND
Core	5.40	0.937	32.10	1.96	ND
Inside	5.40	0.950	35.35	1.74	44.08
L					
Under the rind	5.30	0.924	24.63	1.38	ND
Core	5.29	0.930	29.76	1.99	ND
Inside	5.27	0.948	34.70	1.85	49.39
M					
Under the rind	5.32	0.931	24.01	1.24	ND
Core	5.37	0.926	27.29	1.62	ND
Inside	5.42	0.939	33.47	1.61	50.73
N					
Under the rind	5.36	0.911	25.49	1.57	ND
Core	5.37	0.915	27.49	2.05	ND
Inside	5.42	0.925	28.09	1.94	43.11
O					
Under the rind	5.41	0.879	16.92	1.49	ND
Core	5.48	0.900	25.12	2.05	ND
Inside	5.48	0.909	28.58	2.17	49.01

All brands were made using cow milk

The pH of the Gouda cheese samples ranged from 5.26–6.37. pH was highest in samples removed from under the rind compared with the respective core and inside samples for 11 brands (73%). The sample taken under the rind of brand A had the highest pH overall (6.37). This brand also had the lowest overall pH in the core sample (5.26), leading to a pH difference between the two regions of 1.11; a similar difference of 1.02 was also observed in brand I. All other brands had pH differences between regions of less than 0.53. Overall, no substantial differences in pH values were observed between pasteurized and unpasteurized Gouda cheeses.

The a_w_ of the cheese samples ranged from 0.877 (brand C, under the rind) to 0.957 (brand A, both core and inside). In general, the water activity under the rind was lower than the inside or core samples from the same brand. Similarly to pH, differences in a_w_ values between pasteurized and unpasteurized Gouda cheeses were insignificant. The largest water activity difference between regions of the same brand was 0.030 observed in brand O (0.879 in the sample taken under the rind and 0.909 in the inside sample). A correlation between moisture content and water activity was observed; cheeses which had low moisture contents also had low water activities, which was expected. Salt content for the cheeses ranged from 1.21–2.39%.

### Native microbiota assessment in commercial gouda cheese

**Fig. 1:**
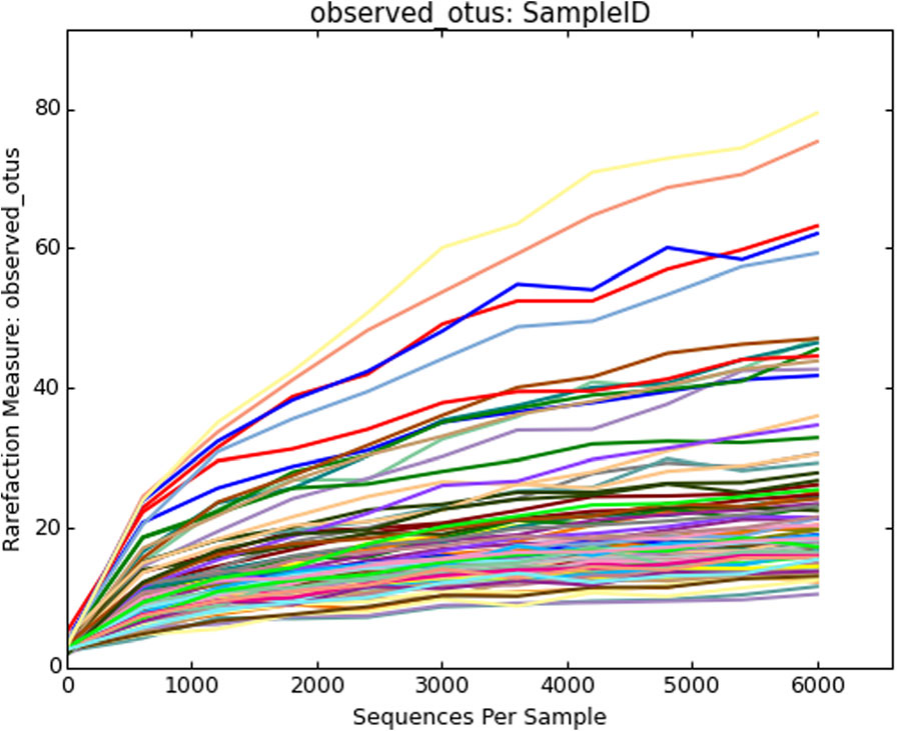
Rarefaction curves of all commercial Gouda cheeses assessed in this study

**Fig. 2:**
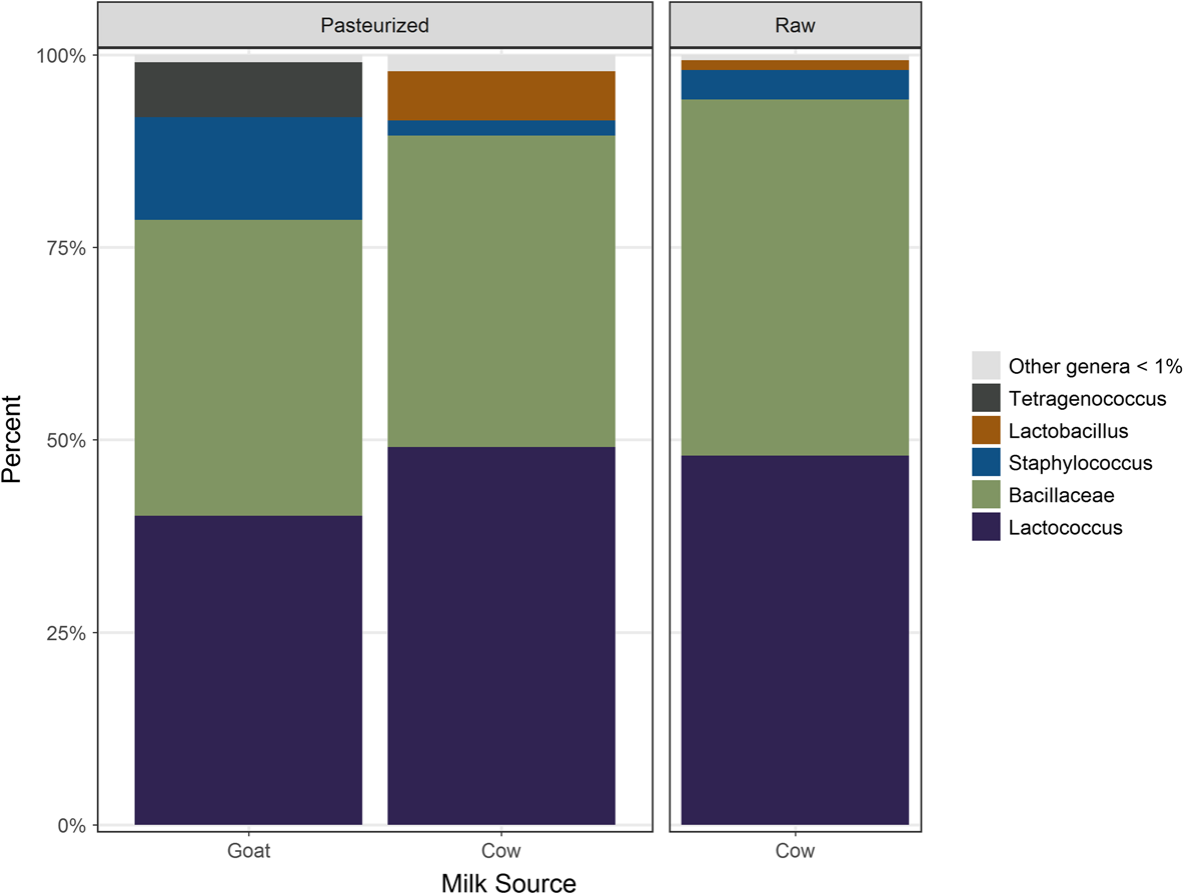
Percentage of bacterial genera in Gouda cheese based on milk pasteurization and source. The reads from all cheese sampling locations (core, inside, just under the rind) were included

Rarefaction curves of all Gouda cheese samples had similar diversity (Fig. 1). All samples displayed similar rarefaction curves in this study. Figure 2 displays the bacterial composition of the pasteurized and unpasteurized Gouda cheeses based on percentage of sequence reads identified at the family or genus levels. Identifications greater than 1% and common to all cheeses included the genera of *Lactococcus* and *Staphylococcus*, and unidentified members of the family *Bacillaceae*. The family *Bacillaceae* included organisms which could not be further identified to genus. *Lactococcus* populations were comparable and ranged from 40.1–49.1%. Bacteria from the family *Bacillaceae* comprised 40.5, 38.5, and 46.3% of the population of pasteurized cow and goat cheese and unpasteurized cow Gouda cheese, respectively. *Staphylococcus* reads were found in low numbers in the three cheese categories: 2.0, 13.4, and 1.3% of the population of pasteurized cow, goat, and unpasteurized cow Gouda cheeses, respectively.

A total of 92, 138, and 120 genus- or family-level identifications were made for pasteurized cow, pasteurized goat, and unpasteurized cow Gouda cheeses, respectively. Eight bacterial genera were identified only in pasteurized cow Gouda cheese and included *Anoxybacillus*, *Curtobacterium*, and *Yersinia*. A total of 28 genera were identified only in the pasteurized goat Gouda cheese in this study and included *Mannheimia*, *Leptotrichia*, *Balneimonas*, *Klebsiella*, and *Pseudoalteromonas*.

### Spatial variability of bacterial genera in commercial gouda cheese

**Fig. 3:**
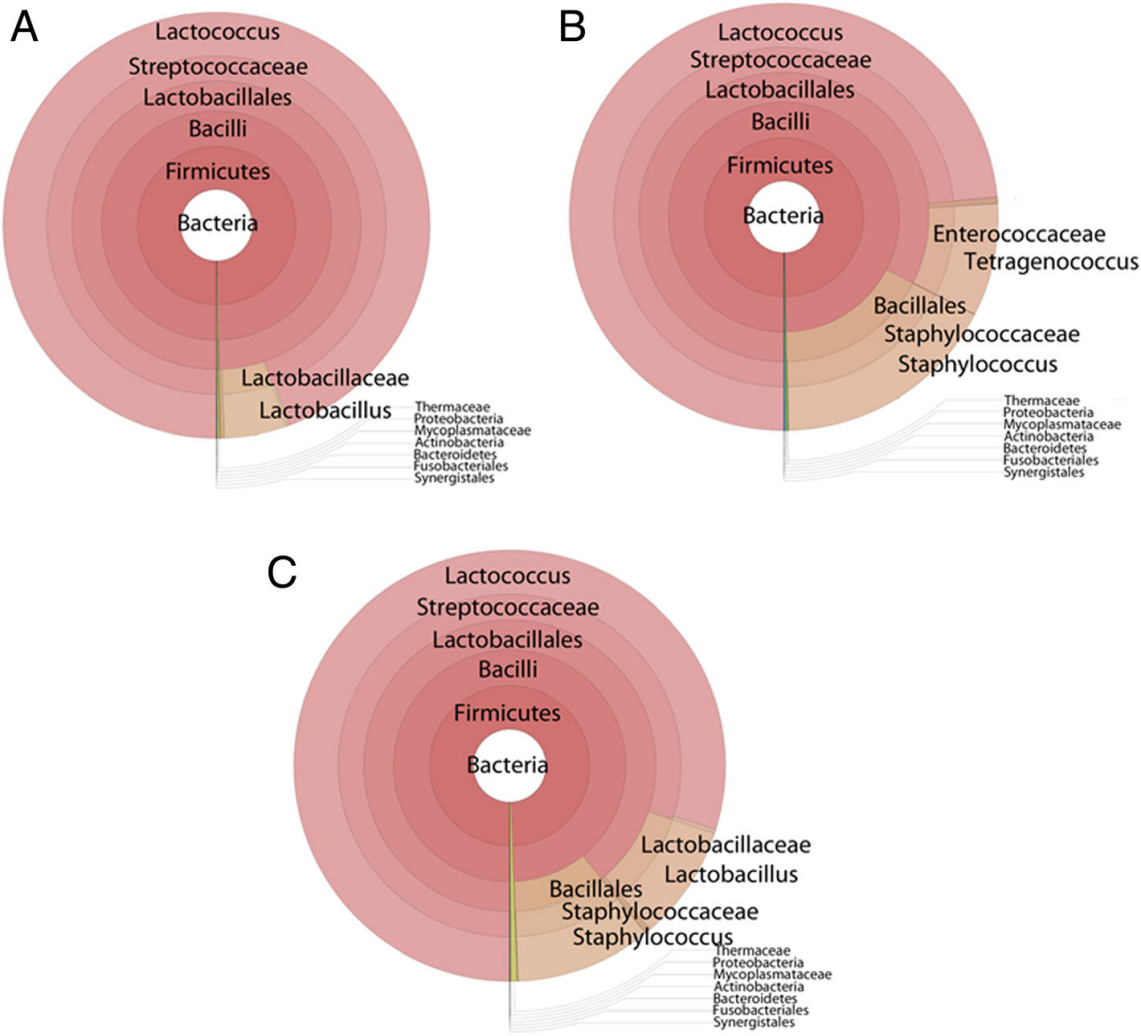
Bacterial kronographs of core (**a**), under the rind (**b**), and inside (**c**) locations of the commercial Gouda cheese

Kronograhs of the bacterial composition of the core, under the rind, and the inside of the commercial Gouda cheeses assessed in this study are presented in Fig. 3. A total of 41 bacterial genera were common to all three locations (core, under the rind, and inside) including *Lactococcus* (55.1, 41.5, and 46.6%), unidentified members of *Bacillaceae* (40.9, 43.1, and 40.6%), *Lactobacillus* (2.8, 0.2, and 5.1%), *Staphylococcus* (0.02, 9.6, and 6.0%), and *Tetragenococcus* (0.004, 4.8, and 0.03%). Overall, the composition of the cores and insides of the Gouda cheeses were more similar to each other based on sequence reads than to the samples taken under the rind. *Lactococcus* and *Lactobacillus* populations were less in the samples taken under the rind. Generally, all the bacterial genera identified in this study were present in all three cheese regions. However, *Megasphaera*, *Caloramator*, and *Hymonella*, were only detected in the cheese cores, and *Anoxybacillus* and *Yaniella*, were only detected in the inside samples. *Brachybacterium*, *Pseudoalteromonas*, *Yersinia*, *Klebsiella*, and *Weissella* were only detected in the samples taken under the rind.

### Influence of aging on the metagenomics of commercial gouda cheese

**Fig. 4:**
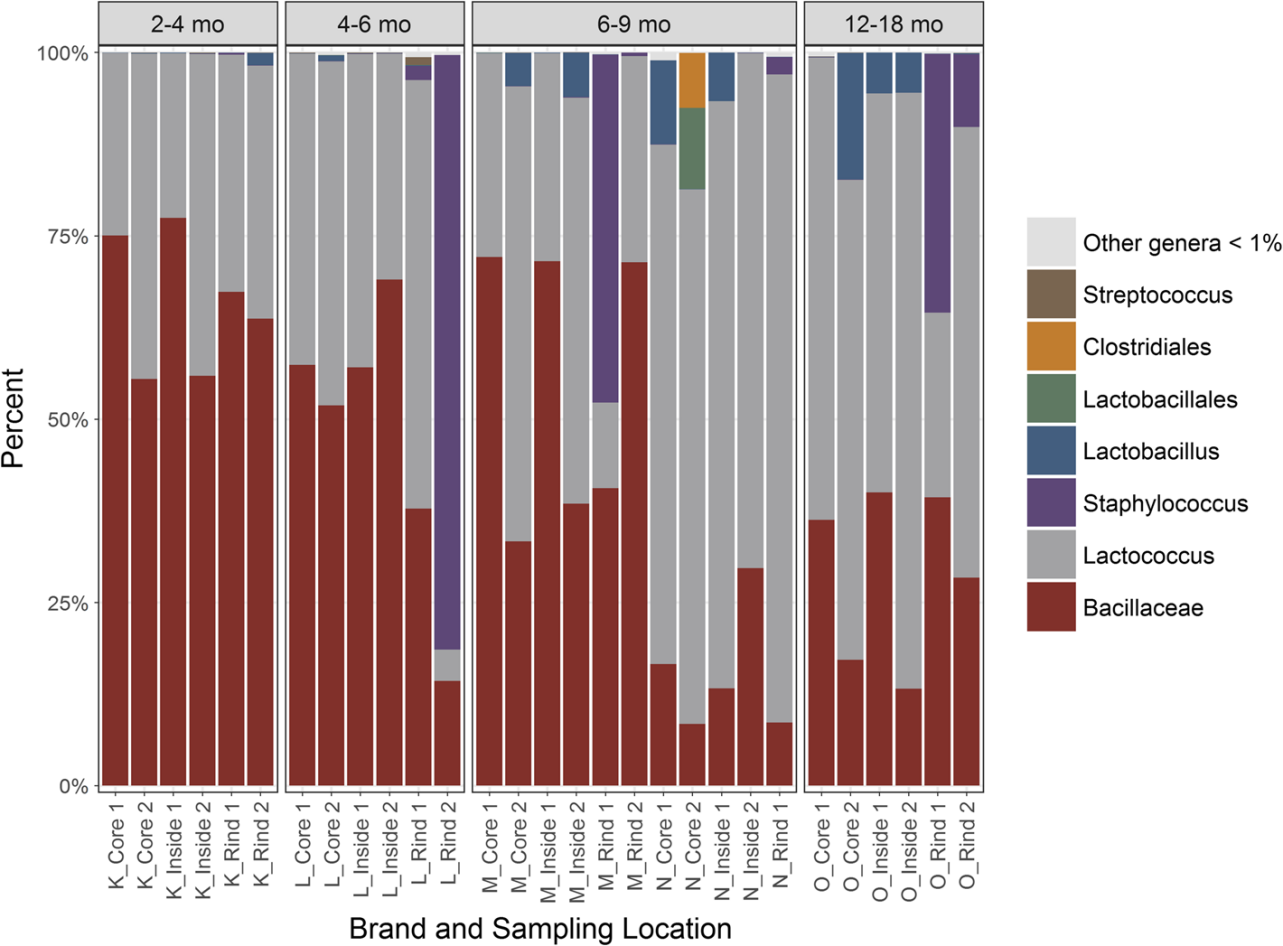
Bacterial genera in commercial unpasteurized Gouda cheese from the same manufacturer based on aging length

Figure 4 depicts the metagenomics of commercial unpasteurized Gouda cheeses from the same manufacturer based on the length of aging (2–4, 4–6, 6–9, or 12–18 months). Unidentified members of *Bacillaceae, Lactococcus, Lactobacillus*, and *Staphylococcus* dominated the populations of the aged Gouda cheeses. *Bacillaceae* sequencing reads decreased during aging and comprised 65.8, 47.9, 36.7, and 29.0% of the population of Gouda cheeses aged for 2–4, 4–6, 6–9, and 12–18 months, respectively. The reverse was observed for *Lactococcus*, where populations increased during aging: this genus comprised 33.7, 37.6, 54.2, and 58.5% of the populations, respectively. *Lactobacillus* and *Streptococcus* populations were 0.3 and 0.4% in the Gouda cheese that was aged for 2–4 months, respectively. In the 12–18 month aged Gouda, the populations were 4.8 and 0.2%, respectively. For the Gouda sample aged 4–6 months, *Staphylococcus* comprised 81.1% in the samples taken under the rind. *Staphylococcus* comprised a lower percentage of total sequencing reads in the Gouda aged for 6–9 or 12–18 months (50.2 or 45.2%, respectively). Less than 1% of the population of the 2–4-month aged Gouda cheese samples taken under the rind was *Staphylococcus*.

Twenty-two and 38 genus-level identifications were observed in the unpasteurized Gouda cheese aged for 2–4 and 12–18 months, respectively (data not shown). A total of 27 out of the 38 identifications in the older Gouda cheese were not found in the younger 2–4 months aged Gouda. Some of the genera identified in the Gouda cheese which was aged longer included *Acidovorax*, *Ralstonia*, *Adhaeribacter*, *Devosia*, *Haemophilus*, and *Neisseria*.

## Discussion

This study assessed physical characteristics of commercially-available pasteurized and unpasteurized Gouda cheeses. According to the FDA Code of Federal Regulations (CFR) [25], the standard of identity of Gouda cheese includes a maximum moisture content of 45% and a minimum fat in solid content of 45%. The Gouda cheeses made with goat milk had higher fat in solid contents than the cheeses made with cow milk, which was expected as goat milk generally contains more fat than cow milk [32]. Four out of the 15 Gouda cheese samples had fat in solid contents which were lower than the 45% minimum specified in the CFR (43.09–44.25%), which was not expected. Also unanticipated was the fact that some of the samples taken under the rind did not have the higher salt contents as compared with the core samples. Intact Gouda cheese wheels are brined in a salt solution, resulting in greater penetration of salt to the outside areas of the cheese, and less penetration of salt to the inner core. This finding, however, may not reflect all Gouda cheeses as only 15 brands were assessed in this study.

Targeted metagenomic sequencing of the 15 brands of commercial Gouda cheese identified two common genera comprising more than 1% of the total sequencing reads among all samples: *Lactococcus* and *Staphylococcus*. In addition, unidentified members of the family *Bacillaceae* were also common to all cheeses. A comprehensive metagenomic study of artisanal semi-hard cheeses, of which Gouda is a member, determined that the overall population of *Lactococcus* was 84.5% based on sequence reads from 31 cheeses [8], which is higher than the result found in the current study (40.1–49.1%). This finding could be a reflection of the starter cultures used to make the cheese or the variation of organisms in the milk. Other studies have determined populations of *Lactococcus* to be 2–22% in different brands of Latin-style cheeses [9] and 49.6% in hard cheeses [8]. *Lactococcus* strains, such as *L. lactis* subspecies *lactis* and *cremoris* are commonly added as part of the starter cultures in cheese manufacture and are responsible for acidification by converting milk lactose into lactic acid. In addition to acidification, bacteria in the genus *Lactococcus* contribute to curd production and the conversion of amino acids into flavoring compounds.

Bacteria from the family *Bacillaceae* comprised 38.5–46.3% of the population of the Gouda cheeses. Many Gram-positive, heterotrophic bacterial genera are part of this family including *Bacillus*, *Salinibacillus*, *Paenibacillus*, *Geobacillus*, and *Lysinibacillus*. These bacteria are found in the milk production chain and are sometimes contaminants in processed cheese [33, 34]. For instance, bacteria in the family *Bacillaceae* comprised less than 10% of the population of Latin-style cheese [9]. The higher percent of *Bacillaceae* in the unpasteurized Gouda cheese in this study (46.3%) may be due to the native microbiota present in the milk used for production. Unpasteurized silo milk contains a greater proportion of *Bacillaceae*, especially when milk is exposed to elevated environmental temperatures [35, 36]. Organisms in this family are often harder to eliminate in the processing environment due to their ability to form biofilms and heat-resistant endospores permitting their resilience to sanitization processes.

*Staphylococcus* reads were found in relatively low numbers in the Gouda cheeses (2.0–13.4%). *Staphylococcus* species, such as *Staphylococcus equorum*, can be used as an additive to the starter culture for certain semi-hard cheeses such as Swiss cheese [37] and are also naturally occurring microorganisms in cheese brines. Interestingly, it has been determined that *S. equorum* possesses anti-listeria properties and some studies have suggested the use of this species as a protective starter culture [38]. *Staphylococcus* has been detected at 0.17% in semi-hard cheeses [8], and at less than 3% in Latin-style cheeses [9]. *Staphylococcus* has been found in high numbers (5–25%) on the surface of certain cheeses, especially early in the aging process and in cheeses made using goat milk [20, 39]. *Staphylococcus* species *epidermidis* and *caprae*, have also been isolated from goat milk [40].

There were a greater number of genus- or family-level identifications observed for the pasteurized goat (*n* = 138) and unpasteurized cow Gouda cheeses (*n* = 120) compared with the pasteurized cow Gouda cheeses (*n* = 92). This is not surprising, as unpasteurized milk has not undergone treatment to eliminate pathogens and reduce the bacterial burden. This is consistent with other studies that have shown unpasteurized cheeses contained a more diverse microbiome than pasteurized cheeses [41]. In this study, 18 genera were identified only in unpasteurized cow Gouda cheese and not in the pasteurized cow or goat Gouda cheeses. Some of the genera identified in the unpasteurized Gouda included *Mycoplasma*, *Ochrobactrum*, *Nocardioides*, *Yaniella*, and *Adhaeribacter*. *Mycoplasma* is a bacterium that can cause mastitis in dairy cattle, and *Ochrobactrum* has been isolated from cow teat skin [42]. *Nocardioides*, *Yaniella*, and *Adhaeribacter* have all previously been identified in unpasteurized milk and cheese [23, 43, 44]; *Yaniella*, a Gram positive coccus in the family *Micrococcaceae*, has been typically found in saline soils and has also been found in cheese rinds [23].

Eight bacterial genera were identified only in pasteurized cow Gouda cheese and included *Anoxybacillus*, *Curtobacterium*, and *Yersinia*. All three genera have previously been isolated from dairy products [45–48]. *Anoxybacillus* is a thermophilic spore-former frequently isolated from whole milk powder and nonfat dry milk and is sometimes used as a hygiene indicator in pasteurized diary manufacture due to its high optimum growth temperature [48]. Unpasteurized milk often contains the potential pathogen *Yersinia enterocolitica* and the organism can be found in curd samples when the milk is used to make cheese. However, in one study, *Y. enterocolitica* has been identified in one out of 265 pasteurized milk samples [47].

A total of 28 genera were identified only in the pasteurized goat Gouda cheese in this study and included *Mannheimia*, *Leptotrichia*, *Balneimonas*, *Klebsiella*, and *Pseudoalteromonas*. *Mannheimia* and *Leptotrichia* may have been part of the goat ecosystem which was transmitted to the milk used in the manufacture of the cheeses. The genus *Mannheimia* is comprised of bacteria responsible for epizootic pneumonia and mastitis in goats, sheep, and cattle [49], while *Leptotrichia* has been isolated from goat foot lesions [50] and are normally found in the human oral cavity [51]. *Balneimonas* has not previously been isolated from cheese, but has been isolated from Suanzhou (Chinese fermented cereal gruel) samples [52]. Bacteria in the genus *Klebsiella*, such as *Klebsiella pneumoniae*, are human pathogens and have also been found to cause spoilage in cheese via gas production leading to early blowing of semi-hard and hard cheeses [53]. Like many other organisms, *Klebsiella* lack thermoresistance, which indicates contamination of the cheeses most likely occurred during or post-manufacture. *Pseudoalteromonas* are mesophilic or psychrophilic marine bacteria that can survive in environments with high salinity. The genera has been identified in soft and semi-hard cow pasteurized and unpasteurized cheeses as well as in cheese rinds [8] and on the surfaces of smear-ripened cheeses [54, 55]. In one study, *Pseudoalteromonas haloplanktis* comprised 17% of the total mapped reads of a smear-ripened cheese as determined using 16S rDNA metagenomics sequencing [54].

Overall, the majority of all the bacterial genera identified in this study were present in all three cheese regions (under the rind, core, inside), however differences were observed in population proportions. In the samples taken under the rind, *Staphylococcus* and *Tetragenococcus* were prevalent (9.6 and 4.8% of the total sequencing reads for all cheeses, respectively). Large populations of *Staphylococcus* on the surfaces of cheeses have been detected previously [23, 56]. *Tetragenococcus*, a moderately halophilic bacterial genus, has previously been detected in unpasteurized hard cheeses and cheese rinds at 0.05 and 0.18%, respectively, but was not detected in soft or semi-hard cheeses [8]. In addition, *Brachybacterium*, *Pseudoalteromonas*, *Yersinia*, *Klebsiella*, and *Weissella* were only detected in the under the rind samples. *Brachybacterium* and *Pseudoalteromonas* are both halophiles, capable of growing in concentrations of salt as high as 15–18% [57, 58]. Therefore, these organisms may have contaminated the cheese during brining. *Yersinia* and *Klebsiella* contain species which are potential human pathogens and are ubiquitous in the environment and could possibly be a post-pasteurization contaminant. *Yersinia* can also grow at refrigeration temperatures and could survive the cheese aging and storage process. Lastly, *Weissella*, a facultative anaerobic lactic acid bacteria in the family *Leuconostocaceae*, was also only identified in the samples taken just under the rind. Although some species of *Weissella* are pathogenic, some species are being studied as potential pro- and prebiotic organisms. This organism has been previously identified in a wide range of habitats including milk and cheese rinds [59], Mexican Cotija cheese [60], and cheese whey [61].

*Megasphaera*, *Caloramator*, and *Hymonella* were only detected in the Gouda cheese cores, and *Anoxybacillus* and *Yaniella* were only detected in the inside samples. The core of a cheese represents an environment that is mainly anaerobic, explaining why the anaerobes *Megasphaera* and *Caloramator* and the facultative anaerobe *Hymonella* were identified in this region. Interestingly, *Megasphaera* is known to be a commensal organism of ruminants and has been identified in unpasteurized ewe milk cheeses [62]. *Anoxybacillus* and *Yaniella*, which were only identified in the inside, were also only present in the cow Gouda cheese samples in this study. This is only the second report of *Yaniella* detected in a food product [23].

In addition to assessing the microflora of Gouda cheese through milk type and spatial variability, this study also examined the differences in microflora based on cheese aging length. Unidentified members of *Bacillaceae, Lactococcus, Lactobacillus*, and *Staphylococcus* dominated the populations of the unpasteurized Gouda cheeses which were aged for 2–4, 4–6, 6–9, or 12–18 months. *Bacillaceae* sequencing reads decreased during aging, whereas the reverse was observed for *Lactococcus*. Lactic acid bacteria, including those of the indigenous microbiota and the added starter cultures, typically comprise most the population of cheese during the aging process [21]. For Swiss and Emmental cheeses, thermophilic lactic acid bacteria derived from the starter culture (such as *Lactobacillus helveticus* and *Streptococcus thermophilus*) are the dominant organisms from the start of aging up to six months [21, 63, 64]. Mesophilic lactic acid bacteria, including *Lactobacillus paracasei* and *L. rhamnosus* also become dominant during aging, especially in cheeses aged for 10–30 months [65].

Interestingly, the population of *Staphylococcus* was not dependent on the length of aging, but rather spatial variation. Most *Staphylococcus* in the aged Gouda was located in samples taken under the rind. For the Gouda sample aged 4–6 months, this genus comprised 81.1% in the samples taken under the rind. However, *Staphylococcus* decreased to 50.2 or 45.2% in Gouda aged for 6–9 or 12–18 months, respectively. Less than 1% of the population of the 2–4-month aged Gouda cheese samples taken under the rind was *Staphylococcus*. The vast differences in these results are likely due to the environmental conditions of aging, personnel handling, and the pasteurization status of the milk used. Large populations of *Staphylococcus* have previously been observed on cheese rinds [23, 56], possibly due to environmental contamination. Furthermore, *Staphylococcus* is presumed to be at a concentration of 2–3 log CFU/mL in unpasteurized milk [21].

Twenty-two and 38 genus-level identifications were observed in the unpasteurized Gouda cheese aged for 2–4 and 12–18 months, respectively. A total of 27 out of the 38 identifications in the older Gouda cheese were not found in the younger 2–4 months aged Gouda. Some of the genera identified in the Gouda cheese which was aged longer included *Acidovorax*, *Ralstonia*, *Adhaeribacter*, *Devosia*, *Haemophilus*, and *Neisseria*. *Acidovorax* and *Ralstonia* are both aerobic Gram-positive plant pathogens [66, 67]. *Acidovorax* has been previously identified as a contaminant of Italian Grana cheese [68], and *Ralstonia* has been detected in unpasteurized milk [43, 69] and can survive high salinity environments. *Adhaeribacter* and *Devosia* are both soil dwelling bacteria and have been previously identified in unpasteurized milk [44, 70]. *Devosia* has also been detected on cow teat skin [42]. *Haemophilus* and *Neisseria*, both genera which contain species of human pathogens, were also only detected in the Gouda cheese that was aged for 12–18 months. However, these genera have not previously been identified in dairy products.

## Conclusions

This study assessed the metagenomics in commercial pasteurized and unpasteurized Gouda cheeses. Overall, the Gouda cheeses assessed were comprised of the same organisms although with different population levels. Some differences were observed between the pasteurized and unpasteurized Gouda cheeses, with more genus-level identifications being made for the unpasteurized cheeses. Twenty-eight bacterial genera were only observed in the goat Gouda cheese, indicating that milk source has vast implications for the resulting microbiome of Gouda cheese. Many other factors can influence the microbiome of Gouda cheese, including spatial variability and length of aging. Aerobic organisms and environmental contaminants were generally identified in outer portions of the Gouda samples. In addition, the length of aging plays an important role in the fate of the microbiome, with an increased level of genus diversity being observed with Gouda cheeses which were aged for longer periods of time. Overall, these results agree with the published literature on cheese microbiomes and provide valuable insights into the microbiome of Gouda cheese. Understanding the metagenomics of Gouda cheese is useful in improving sensory characteristics, extending shelf-life, and improving product quality and safety.

## Methods

### Gouda cheeses and sampling locations

To determine the microbiota of commercially prepared, domestic and imported, pasteurized (*n* = 8) and unpasteurized (*n* = 7) Gouda cheese samples were obtained from three retailers in Wisconsin and Illinois. The fifteen different brands were labelled A through O and stored at 5 °C prior to analysis. Detailed information on the Gouda cheeses used in this study is listed in Table 3. All cheeses were purchased as wedges cut from a larger cheese wheel and were either waxed or wrapped in plastic. Three main samples were extracted from each cheese wedge. One sample was taken from just under the rind of the cheese. A corer was used to remove a second sample labeled as “core” from the middle of the cheese wedge. The top and bottom portions of the core were subsequently removed. A third sample consisted of the thinnest portion of the wedge which represents the innermost regions of the complete wheel from which the cheese wedge was originally cut. This sample was labeled as “inside”.

**Table 3:** Commercial pasteurized and unpasteurized Gouda cheeses analyzed in this study

Brand	Milk used	Animal	Aging length	Listed ingredients	Specified geographic region
A	Pasteurized	Cow	NA	Part-skim milk, salt, culture, microbial rennet, annatto	U. S.
B	Pasteurized	Cow	NA	Milk, cultures, salt, enzymes, carotene	Netherlands
C	Pasteurized	Cow	NA	Milk, salt, culture, rennet, annatto	Netherlands
D	Pasteurized	Cow	NA	Milk, salt, culture, vegetarian rennet	Netherlands
E	Pasteurized	Cow	NA	Milk, salt, rennet, beta carotene	Netherlands
F	Pasteurized	Goat	NA	NA	NA
G	Pasteurized	Goat	NA	NA	Netherlands
H	Pasteurized	Goat	NA	Milk, salt, vegetable rennet	Netherlands
I	Unpasteurized	Cow	NA	Milk, cultures, enzymes, salt	U. S.
J	Unpasteurized	Cow	NA	Milk, salt, cultures, rennet	NA
K	Unpasteurized	Cow	2–4 mo	Milk, cultures, enzymes, salt	U. S.
L	Unpasteurized	Cow	4–6 mo	Milk, cultures, enzymes, salt	U. S.
M	Unpasteurized	Cow	6–9 mo	Milk, cultures, enzymes, salt	U. S.
N	Unpasteurized	Cow	6–9 mo	Milk, cultures, vegetable enzymes, salt	U. S.
O	Unpasteurized	Cow	12–18 mo	Milk, cultures, enzymes, salt	U. S.

NA, this information was not available as these samples were repackaged by a local retailer

Brands K, L, M and O were from the same manufacturer

### Cheese property characteristics

To assess variations in the physical property characteristics of the Gouda cheeses, 20 g samples from each of the three regions (just under the rind, core, inside) were subjected to moisture, salt, fat, pH, and a_w_ analysis. Single or duplicate samples were designated for each assay depending on the amount of cheese available. Each cheese sample was equilibrated at ambient temperature for 30 min and then grated to facilitate analysis. Moisture content was determined by heating 3 g of cheese to 101 °C in an OHAUS MB45 moisture analyzer (OHAUS Corporation, Parsippany, NJ) until no further weight changes due to moisture loss was detected for 90 s. Fat content was determined using the Babcock method for fat analysis of cheese products [71] utilizing sulfuric acid to digest 9 g of cheese. Water activity (a_w_) was measured by testing 0.5–0.7 g of cheese using an AQUA Lab, METRFood 4TEV Water Activity Meter (Pullman, WA). Salt content was determined by homogenizing 5 g of cheese in 98 mL of 60 °C deionized water for 3 min at 180 rpm using a Seward 3500 stomacher (Seward Laboratory Systems Inc., Davie, FL) and measuring the salt content of the homogenate using a Chloride Analyzer M926 (Nelson-Jameson Inc., Marshfield, WI) following the manufacturer’s instructions. The pH of the cheese samples was determined using a Eutech pH Spear (Oakton Instruments, Vernon Hills, IL) and an Extech pH meter (Extech Instruments, Watham, MA).

### Total DNA extraction

DNA was extracted from duplicate 1 g samples collected from each of three regions (just under the rind, core, inside) for each of the 15 Gouda cheeses. Extractions were conducted using the PowerFood Microbial DNA Isolation Kit (MO BIO Laboratories, Carlsbad, CA) according to the manufacturer’s instructions. DNA was quantified using the Qubit dsDNA BR Assay Kit (Invitrogen, Carlsbad, CA) according to the manufacturer’s instructions, and stored at − 20 °C prior to PCR.

### PCR amplification of 16S rRNA genes

For each DNA sample (*n* = 90), 3 ng was used as the PCR template and one of four 16S rDNA primer pairs was used to target the V4 region (Table 4). Primer pairs differed by a nucleotide shift and were randomly assigned to samples equally for use in amplification of the targeted 16S rDNA region. PCR was conducted using Omni Klentaq (DNA Polymerase Technology, St. Louis, MI), PCR Enhancer Cocktail 1 (PEC-1, DNA Polymerase Technology), and 10 μM of each forward and reverse primer. Cycling conditions consisted of 94 °C for 2 min, 25 cycles of 94 °C for 40 s, 56 °C for 15 s, and 68 °C for 40 s, followed by 68 °C for 5 min. A negative control consisting of reagents without template DNA was also included in the PCR assay. Amplicons were analyzed using agarose gel electrophoresis, purified using Ampure XP beads (Beckman-Coulter, Indianapolis, IN), and quantified using the Qubit DNA BR Assay kit (Invitrogen) in conjunction with a Qubit 2.0.

**Table 4:** Primer pairs used in this study

Set	Primer	Sequence (5' to 3')
1	26F1a	TCGTCGGCAGCGTCAGATGTGTATAAGAGACAGDAGAGTTTGATCMTGGCTCAG
	534R1	GTCTCGTGGGCTCGGAGATGTGTATAAGAGACAGTMTTACCGCGGCNGCTGGCAC
2	26F2a	TCGTCGGCAGCGTCAGATGTGTATAAGAGACAGADAGAGTTTGATCMTGGCTCAG
	534R2	GTCTCGTGGGCTCGGAGATGTGTATAAGAGACAGCTMTTACCGCGGCNGCTGGCAC
3	26F3a	TCGTCGGCAGCGTCAGATGTGTATAAGAGACAGGCDAGAGTTTGATCMTGGCTCAG
	534R3	GTCTCGTGGGCTCGGAGATGTGTATAAGAGACAGACTMTTACCGCGGCNGCTGGCAC
4	26F4a	TCGTCGGCAGCGTCAGATGTGTATAAGAGACAGGGCDAGAGTTTGATCMTGGCTCAG
	534R4	GTCTCGTGGGCTCGGAGATGTGTATAAGAGACAGGACTMTTACCGCGGCNGCTGGCAC

### Library construction and sequencing

The 16S rDNA gene fragments were indexed using the Nextera XT Kit (Illumina, San Diego, CA) according to the Nextera DNA Sample Preparation Guide (Document#15027987) with some modifications. Index adapter pairs were chosen using Illumina Experiment Manager and the Nextera Low Plex Pooling Guidelines. Each index PCR reaction contained 5 μl of designated i7 and i5 adapter, 10 μl of Omni Klentaq, 20 μl of template DNA (7.5 ng/μl), and 10 μl of ultrapure water for a total reaction volume of 50 μl. The index PCR was cycled according to the Nextera DNA Sample Prep Guide, and the libraries were cleaned up using Ampure XP beads. The sequencing libraries were quantified using the Qubit 2.0 along with the Qubit DNA HS Assay kit, and the quality was assessed on a Bioanalyzer 2100 using the DNA 1000 kit (Agilent, Santa Clara, CA). Indexed libraries were normalized to 2 nM using 10 mM Tris-HCl, 0.1% Tween 20, pH 8.5, and then pooled. The concentration of the pooled 2 nM library was again assessed using a Qubit 2.0. The normalized, pooled 2 nM library was denatured using 0.1 N NaOH and diluted to 10 pM using pre-chilled HT1 buffer supplied in the Nextera XT kit (Illumina). Meanwhile, a 12.5 pM PhiX library was prepared and denatured according to the MiSeq System Denature and Dilute Libraries Guide (Document #15039740v01). The denatured PhiX library was spiked (10%) into the 10 pM denatured, indexed library, which was subsequently sequenced using MiSeq version 3 chemistry.

### 16S rDNA amplicon sequence analysis

Paired-end sequence reads were analyzed as previously described [72]. Sequence counts were rarefied to 6000 sequences for each independent sample.

### Accession numbers

Metagenomic sequence data has been deposited to the SRA of NCBI under Bioproject PRJNA382370, Biosamples SAMN06705955–6046.
